# Quantitative proteomic tools for analyzing histone modifications

**DOI:** 10.1186/1756-8935-6-S1-O11

**Published:** 2013-03-18

**Authors:** Benjamin A Garcia

**Affiliations:** 1 University of Pennsylvania School of Medicine, USA

## 

Histones are small proteins that package DNA into chromosomes, and a large number of studies have showed that several single post-translational modification sites on the histones are associated with both gene activation and silencing. Nevertheless, what type of effect distinct combinations of simultaneously occuring histone modifications (Histone Codes or patterns) have upon cellular events is poorly understood. The main reason for this lack of knowledge is that robust high-throughput methods for quantitative characterization or even qualitative identification of combinatorial Histone Codes by any standard biological, immunological or physical technique do not exist. We plan to specifically address this deficiency by developing novel mass spectrometry based proteomic methods and accompanying bioinformatics to quantitatively characterize molecular level descriptions of combinatorial Histone Codes, and apply these methods to study how these dynamic Histone Codes influence gene expression under different biological conditions. Here we present initial proteomics data that describes: (i) high-throughput comparison of histone modifications from multiple cellular states (ii) developing mass spectrometry methods for quantitative tracking of combinatorial Histone Codes (iii) monitoring *in vivo* Histone Code dynamics, and (iv) investigating the role of Histone Code interpreting proteins in recognizing distinct Histone Codes. Ultimately, we will work towards the goal of taking any defined part of the genome and accurately quantifying the Histone Codes, detecting all the non-histone proteins that reside on these distinct pieces of chromatin, and then mapping this proteomic data back to specific genomic locations, therefore taking a proteomic snapshot of what that chromosome landscape looks like during any nuclear event. These studies in combination with biological experiments will help provide a systems biology outlook on gene expression that will lay down the basic scientific foundation to advance several applications, such as stem cell reprogramming and cancer progression.

